# Identification of antipsychotic drug fluspirilene as a potential anti-glioma stem cell drug

**DOI:** 10.18632/oncotarget.22904

**Published:** 2017-12-04

**Authors:** Yu Dong, Takuya Furuta, Hemragul Sabit, Tomohiro Kitabayashi, Shabierjiang Jiapaer, Masahiko Kobayashi, Yasushi Ino, Tomoki Todo, Lei Teng, Atsushi Hirao, Shi-Guang Zhao, Mitsutoshi Nakada

**Affiliations:** ^1^ Department of Neurosurgery, Graduate School of Medical Science, Kanazawa University, Kanazawa, Japan; ^2^ Department of Pathology, Kurume University School of Medicine, Kurume, Japan; ^3^ Division of Molecular Genetics, Cancer Research Institute, Kanazawa University, Kanazawa, Japan; ^4^ Laboratory of Innovative Cancer Therapy, Institute of Medical Science, University of Tokyo, Tokyo, Japan; ^5^ Department of Neurosurgery, The First Affiliated Hospital of Harbin Medical University, Harbin, People’s Republic of China

**Keywords:** glioma stem cell, glioblastoma, drug screening, drug repositioning

## Abstract

Glioma stem cell (GSC)-targeted therapy is expected to be one of the most innovative approaches to treat patients with glioblastoma (GBM). A number of the drugs that restrain the signaling pathway essential for GSC maintenance have been under clinical trials. Here, we identified fluspirilene, a traditional antipsychotic drug, as a GSC-targeting agent, selected from thousands of existing drugs, and investigated its therapeutic effects against GBM with the purpose of drug repositioning. To develop novel therapeutics targeting GSCs, we initially screened drug libraries for small-molecule compounds showing a greater efficacy, compared to that of controls, in inhibiting the proliferation and survival of different GSC lines using cell proliferation assay. Drugs already reported to show therapeutic effects against GBM or those under clinical trials were excluded from subsequent screening. Finally, we found three drugs showing remarkable antiproliferative effects on GSCs at low concentrations and investigated their therapeutic effects on GSCs, glioma cell lines, and in a GBM mouse model. Of the three compounds, fluspirilene demonstrated a significant inhibitory effect on the proliferation and invasion of glioma cells as well as in the model mice treated with the drug. These effects were associated with the inactivation of the signal transducer and activator of transcription 3 (STAT3). Redeveloping of fluspirilene is a promising approach for the treatment of GBM.

## INTRODUCTION

Glioblastoma (GBM) is one of the most malignant primary brain tumors in adults. Despite major advances in the diagnosis and treatment of cancer in recent years, GBM is still rarely curable, and most patients diagnosed with GBM die within 2 years. Each GBM tumor is composed of heterogeneous cell populations, including those with stem cell properties, termed glioma-initiating cells or glioma stem cells (GSCs) [1]. GSCs are characterized by a highly invasive nature and resistance to chemo- and radiotherapy [2]. Accumulating evidence suggests that targeting GSCs provides considerable benefits in experimental settings [3, 4]. However, none of the agents inhibiting the signaling pathway of GSCs has been approved for use in the clinic.

Development of new drugs is a costly and laborious process with a high rate of failure. Drug repositioning or drug repurposing refers to the identification of new indications for already approved drugs, offering promising options to overcome the problems of drug development [5]. In the past, several drugs were repurposed, including the anti-inflammatory agent aspirin for secondary prophylaxis of cerebrovascular ischemic stroke and cardiovascular disease [6, 7], a chemotherapeutic agent against breast cancer, raloxifene, for the prophylaxis of osteoporotic fractures [8], the sleep-inducing agent thalidomide for chemotherapy against multiple myeloma [9], the angina pectoris medication sildenafil for erectile dysfunction [10], and azidothymidine, which failed as a chemotherapeutic agent, for anti-human immunodeficiency virus (HIV) therapy [11].

In the present study, we identified fluspirilene, a traditional antipsychotic drug, as a novel therapeutic agent against GBM. We selected fluspirilene from 1,301 existing compounds and examined its therapeutic effects both *in vitro* and *in vivo* for the purpose of drug repositioning. Additionally, the antitumor effect of this drug was revealed to attenuate the signal transducer and activator of transcription (STAT) 3 activity. STAT3 is an important transcription factor for many cytokines and growth factor receptors, and it is associated with the maintenance of cancer stem cells [12–17]. Strong activation of STAT3 by epidermal growth factor (EGF), platelet-derived growth factor, transforming growth factor beta (TGFβ), and interleukin-6 (IL-6) serves as a crucial signal for the maintenance of GSCs and treatment resistance in GBM [12, 14]. STAT3 is involved in a radiation-induced proneural-to-mesenchymal transition [13]. Consequently, targeting STAT3-related signaling pathways is expected to be a promising therapeutic approach to overcoming this refractory disease. Some clinical trials to develop STAT3-targeting drugs against malignant tumors other than glioma were conducted [18–20].

## RESULTS

### Potential candidate compounds against GBM

**Figure 1:**
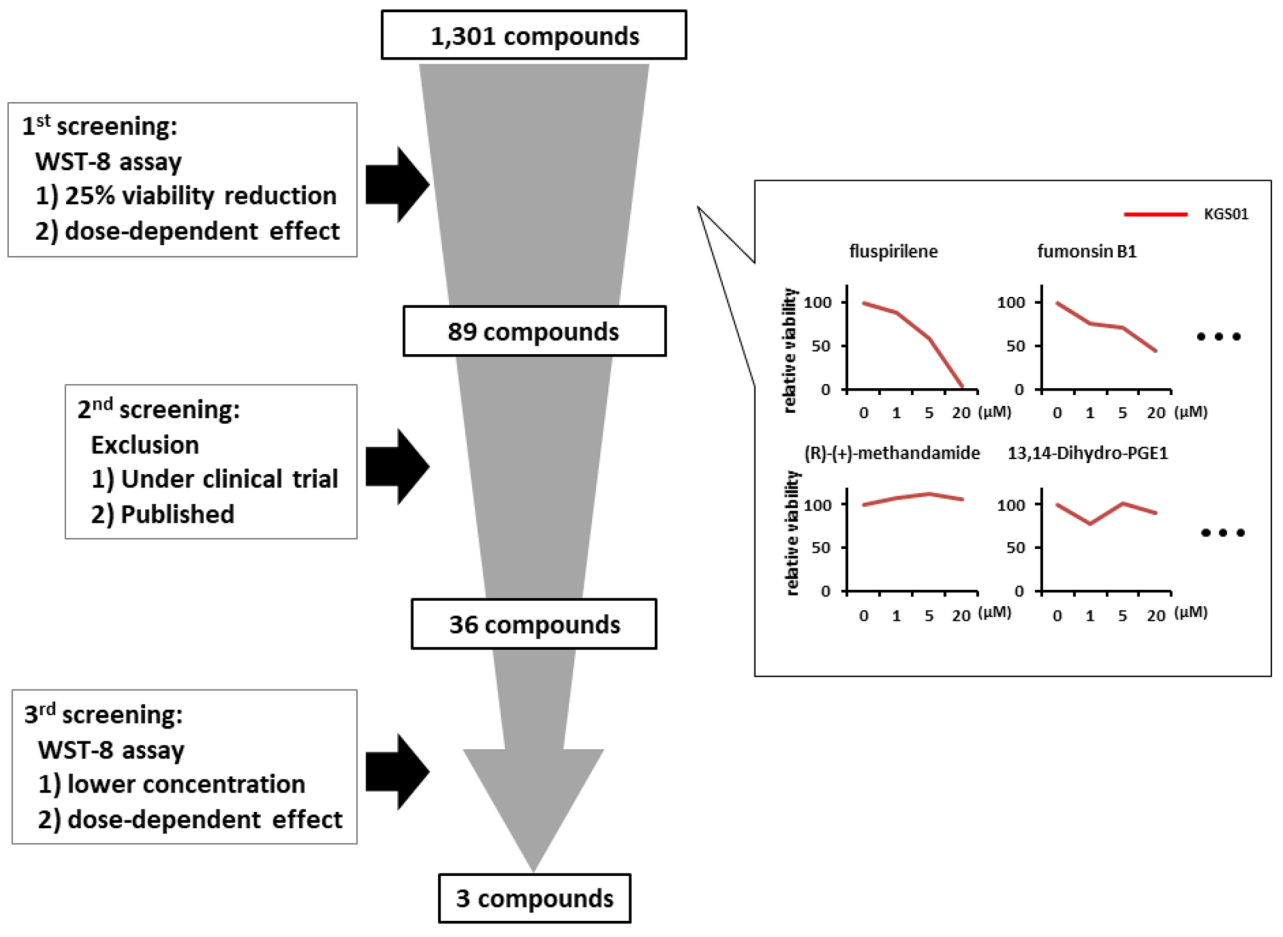
Schematic representation of the drug screening procedure A total of 1,301 compounds from five libraries were screened by a three-step procedure. First, compounds (1, 5, 20 μM) exhibiting 25% or more reduction in the cell viability, measured by the WST-8 assay, were selected. Second, drugs with an already reported therapeutic potential against GBM or those under clinical trials were excluded. Third, drugs attenuating the cell viability and sphere-forming capacity at lower concentrations (0.2, 0.5, 1 μM) were selected. Some examples showing efficacy or inefficacy of 1st screening were presented in the right panels.

Of the 1,301 compounds screened, 89 compounds showed various degrees of viability inhibition of GSCs, as determined by the WST-8 cell proliferation assay during initial screening (Figure 1). After we excluded the drugs that are under clinical trials for GBM or have already been reported to show effects on GBM cells, 36 compounds were identified during a second round of screening. Among those, three drugs were selected, which exhibited strong inhibitory effects on the GBM cell viability at lower concentrations (Figure 1). Of the 3 compounds, fluspirilene (8-[4,4-bis(4-fluorophenyl)butyl]-1-phenyl-1,3,8-triazaspiro [4, 5] decan-4-one) was selected because of its novelty and efficacy in the treatment of GBM cells (Figure 1) and the ability to penetrate blood–brain barrier (BBB) [21]. Fluspirilene is a potent diphenylbutylpiperidine antipsychotic drug that has been used for the treatment of schizophrenia for many years. The other two compounds are being investigated as next candidates.

### Attenuation of stemness and proliferation of GSCs by fluspirilene

**Figure 2:**
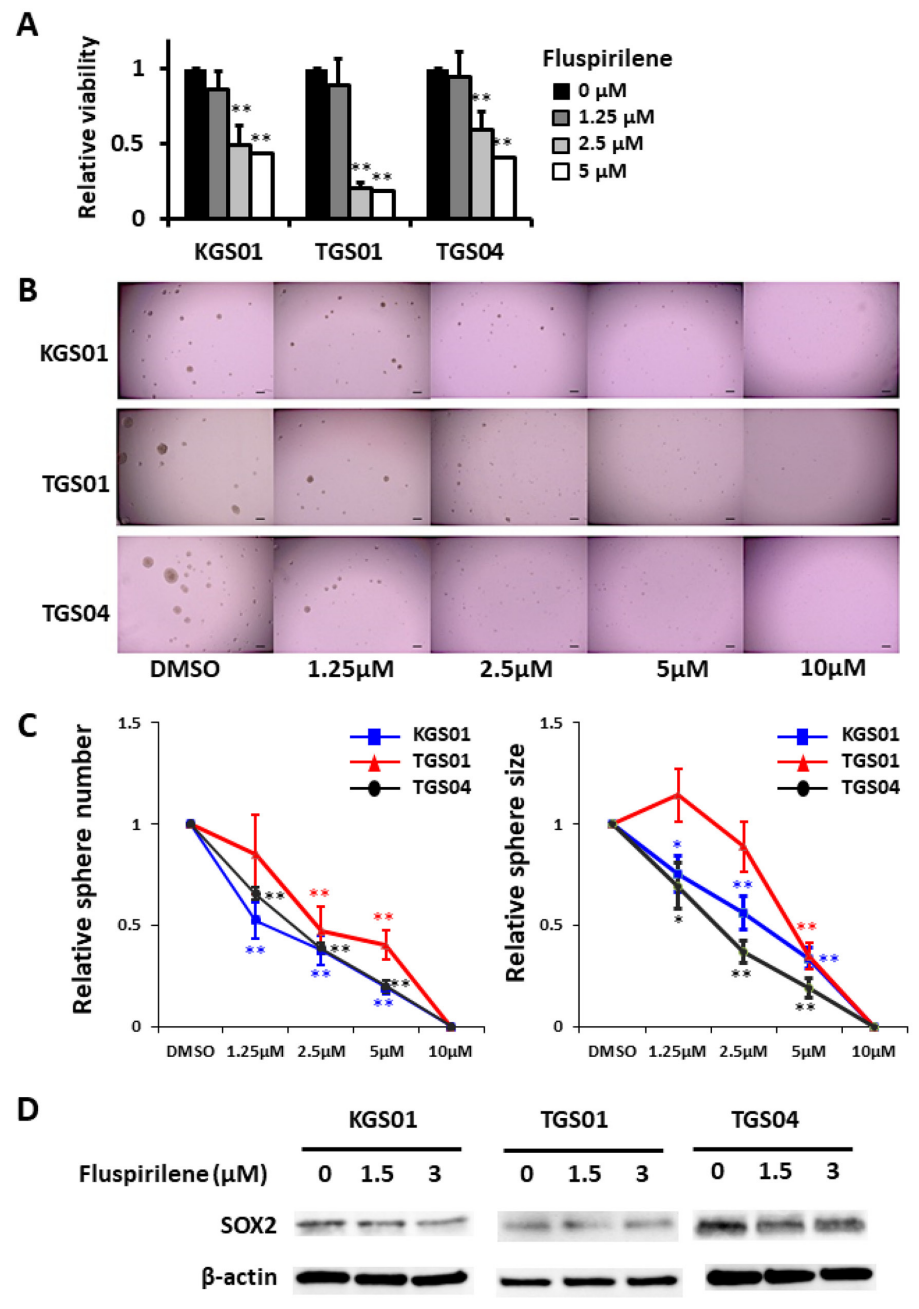
Fluspirilene inhibited viability and suppressed sphere-forming ability of three stem cell lines in a dose-dependent manner by down-regulating SOX2 **(A)** Viability of GSCs was assessed by the WST-8 assay 48 h after the treatment with fluspirilene at various concentrations. ^**^p < 0.01. **(B)** Micrographs show representative tumorspheres formed by relatively sensitive KGS01, TGS01, and TGS04 cells after 7 days of fluspirilene treatment. *Scale bar* = 200 μm. **(C)** The sphere numbers and sizes dramatically decreased in sensitive lines with 1.25 μM or 2.5 μM fluspirilene treatment. The sphere numbers strongly decreased in GSCs at fluspirilene concentrations of more than 2.5 μM. Sphere formation by all three GSC lines was diminished with 10 μM fluspirilene. The sphere sizes of GSCs were significantly reduced in a dose-dependent manner. ^*^p < 0.05, ^**^p < 0.01. **(D)** Fluspirilene attenuated the expression of SOX2 in dose dependent manner. β-actin was used as a loading control.

The WST-8 assay showed that fluspirilene decreased the viability of all three GSC lines in a dose-dependent manner (Figure 2A). At concentrations of 2.5 and 5 μM, fluspirilene exhibited 48.7% and 43.7% reduction in KGS01 (*p* < 0.01), 20.5% and 18.1% reduction in TGS01 (*p* < 0.01), and 59.2% and 40.8% reduction in TGS04 (*p* < 0.01) cell viability, respectively, but not significantly at the lowest concentration of 1.25 μM.

Fluspirilene also decreased the sphere formation by all three GSC lines in a dose- dependent manner (Figure 2B, 2C). The sphere numbers were reduced in KGS01 and TGS04 cultures at all tested concentrations of fluspirilene, while those in the TGS01 culture were reduced at fluspirilene concentrations of 5 μM and more. Additionally, fluspirilene at concentrations above 1.25 μM significantly reduced the sphere sizes for KGS01 and TGS04, while those for TGS01 were reduced at concentrations above 2.5 μM of fluspirilene. Notably, the sphere formation was completely blocked by 10 μM fluspirilene in all three cell lines (Figure 2C). The reduced stem cell properties were associated with suppression of SRY (sex determining region Y)-box (SOX) 2 expression by fluspirilene (Figure 2D). These data indicate that fluspirilene is effective in controlling the stem cell phenotype.

### Effect of fluspirilene on proliferation and invasion of GBM cells and GSCs

**Figure 3:**
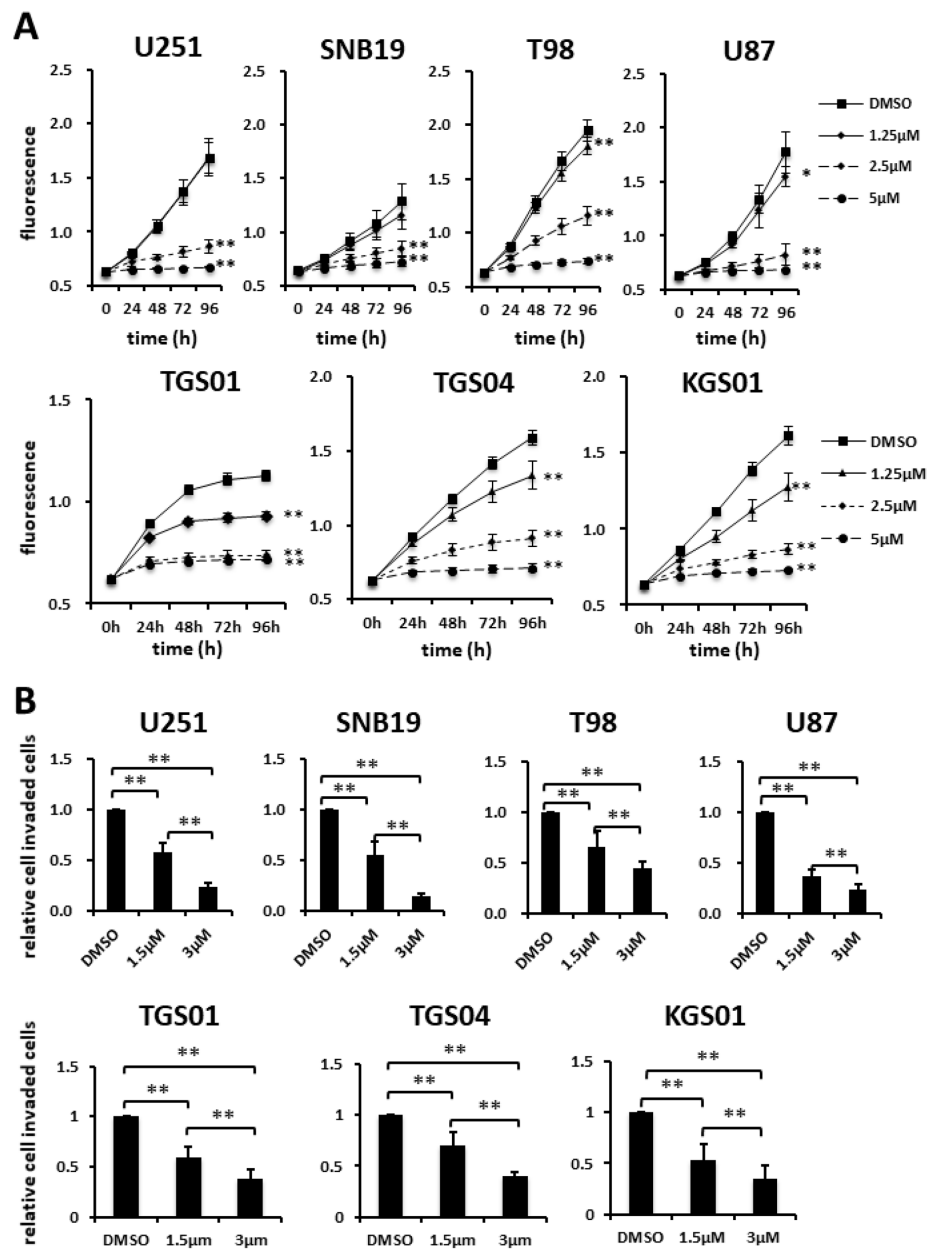
Effects of fluspirilene on proliferation and invasion of GBM cells **(A)** Cells were treated with fluspirilene at 1.25, 2.5, and 5 μM, and viability curves of GBM cell lines were analyzed in the presence and absence of fluspirilene. The plate was read on a plate reader at the indicated time points. **(B and C)** Cells were treated with fluspirilene at 1.5 and 3 μM. Cells invading through a Matrigel-coated Transwell chamber were scored in the presence and absence of fluspirilene for 8 h. The mean numbers of cells and standard deviations were calculated for eight high-power microscopic fields. ^*^p < 0.05, ^**^p < 0.01.

When treating malignant glioma, it is reasonable to expect that the drug will inhibit not only GSCs but also differentiated tumor cells. Thus, we evaluated the efficacy of fluspirilene against both glioma cell lines and GSC lines. Fluspirilene demonstrated the inhibition of proliferation of T98, U87 and all GSC lines at 1.25, 2.5, and 5 μM, while it inhibited the proliferation of U251 and SNB19 at 2.5 and 5 μM, as determined by the Alamar blue assay (Figure 3A). The Transwell assay showed that fluspirilene inhibited the invasion of GBM cells and GSCs to extracellular matrix in a dose-dependent manner (Figure 3B). The numbers of invaded GBM cells were reduced by 55.4% and 14.8% in SNB19 (*p* < 0.01), 37.1% and 23.2% in U87 (*p* < 0.01), 58.0% and 23.9% in U251 (*p* < 0.01), and 66.5% and 45.2% in T98 (*p* < 0.01) by 1.5 and 3 μM fluspirilene, respectively (Figure 3B). The numbers of invaded GSCs were reduced by 59% and 38% in TGS01 (*p* < 0.01), 70.5% and 39.6% in TGS04 (*p* < 0.01), 52.7% and 35% in KGS01 (*p* < 0.01) by 1.5 and 3 μM fluspirilene, respectively (Figure 3B). Taken together, fluspirilene can be considered a potential therapeutic agent against GBM composed of GSCs and differentiated tumor cells.

### Inhibitory effect of fluspirilene on STAT3

**Figure 4:**
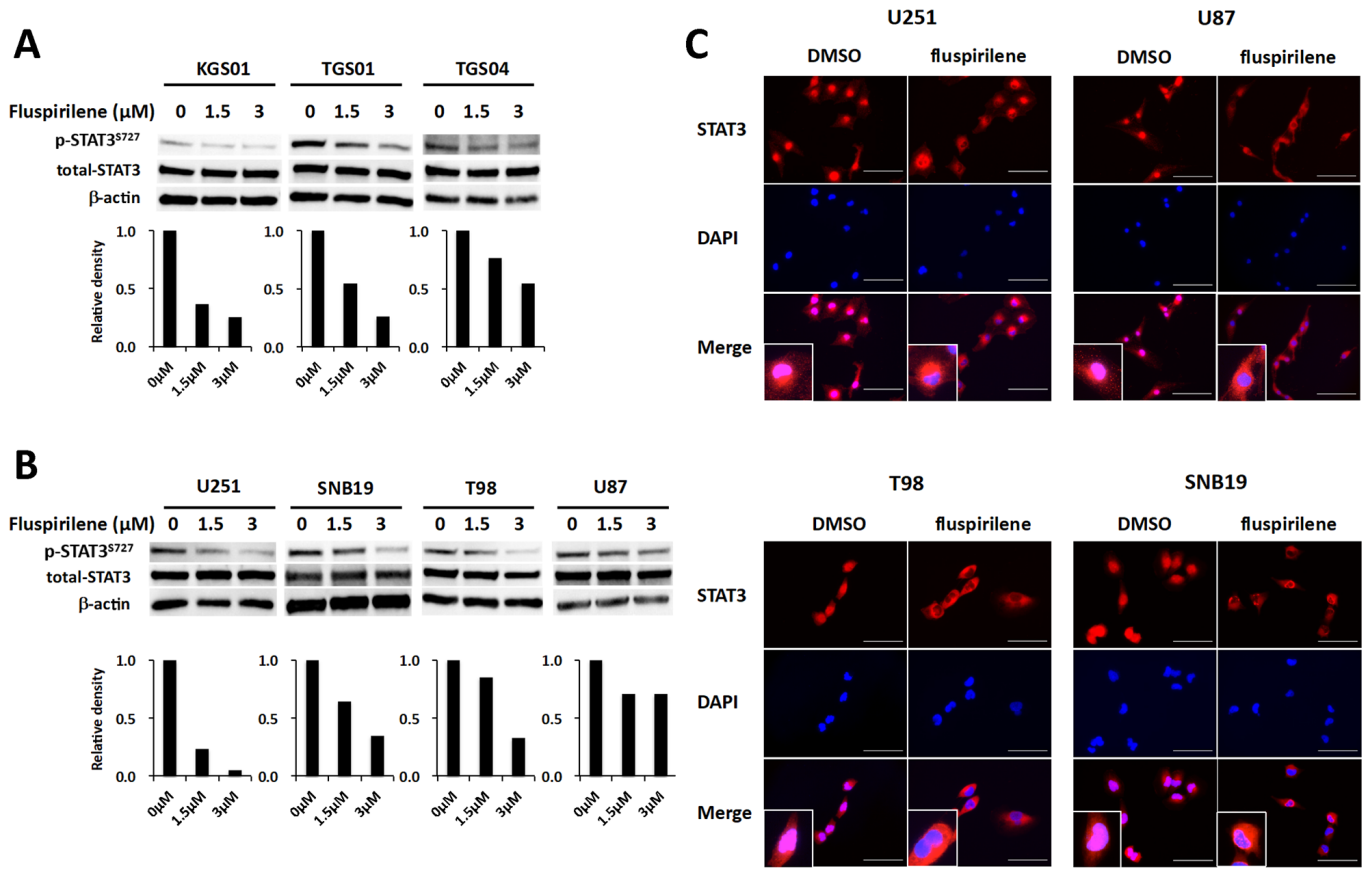
Inhibition of STAT3 activity and modulation of STAT3 translocation into the nucleus by fluspirilene **(A)** Changes in the levels of STAT3 phosphorylated at serine 727 residue [p-STAT3 (S727)] and total STAT3 were analyzed by western blotting in GSCs and GBM cells following the treatment with fluspirilene for 48 h. β-actin was used as a loading control. The bar graphs below the blots show relative levels of p-STAT3 (S727) quantified by densitometry and normalized to those of total STAT3. **(B)** Nuclear content of STAT3 reduced in GBM cell after 1.5 µM of fluspirilene treatment. STAT3 (red), DAPI (blue). *Scale bar* 100μm.

We analyzed various signal transduction pathways characteristic of GBM or other malignant tumors, such as phosphoinositide 3-kinase (PI3K)/protein kinase B (AKT), Janus kinase (JAK)/STAT, cyclin-dependent kinase 2 (CDK2), mouse double minute 2 homolog (MDM2), and RAF kinase/mitogen-activated protein kinase (MAPK) kinase (MEK)/extracellular signal-regulated kinase (ERK) pathways. Consequently, we identified fluspirilene to be a STAT3 inhibitor by monitoring the level of STAT3 phosphorylated at the serine 727 residue [p-STAT3 (S^727^)], the active form of STAT3. Western blot analysis showed that fluspirilene attenuated the expression level of p-STAT3 (S^727^) in a dose- dependent manner both in GSCs (Figure 4A) and glioma cell lines (Figure 4B). Other than STAT3, none of the protein molecules analyzed was significantly altered by fluspirilene (Supplementary Figure 2). In addition, the inhibition of STAT3 phosphorylation coincided with reduced translocation of STAT3 proteins from the cytoplasm to the nucleus (Figure 4C).

**Figure 5:**
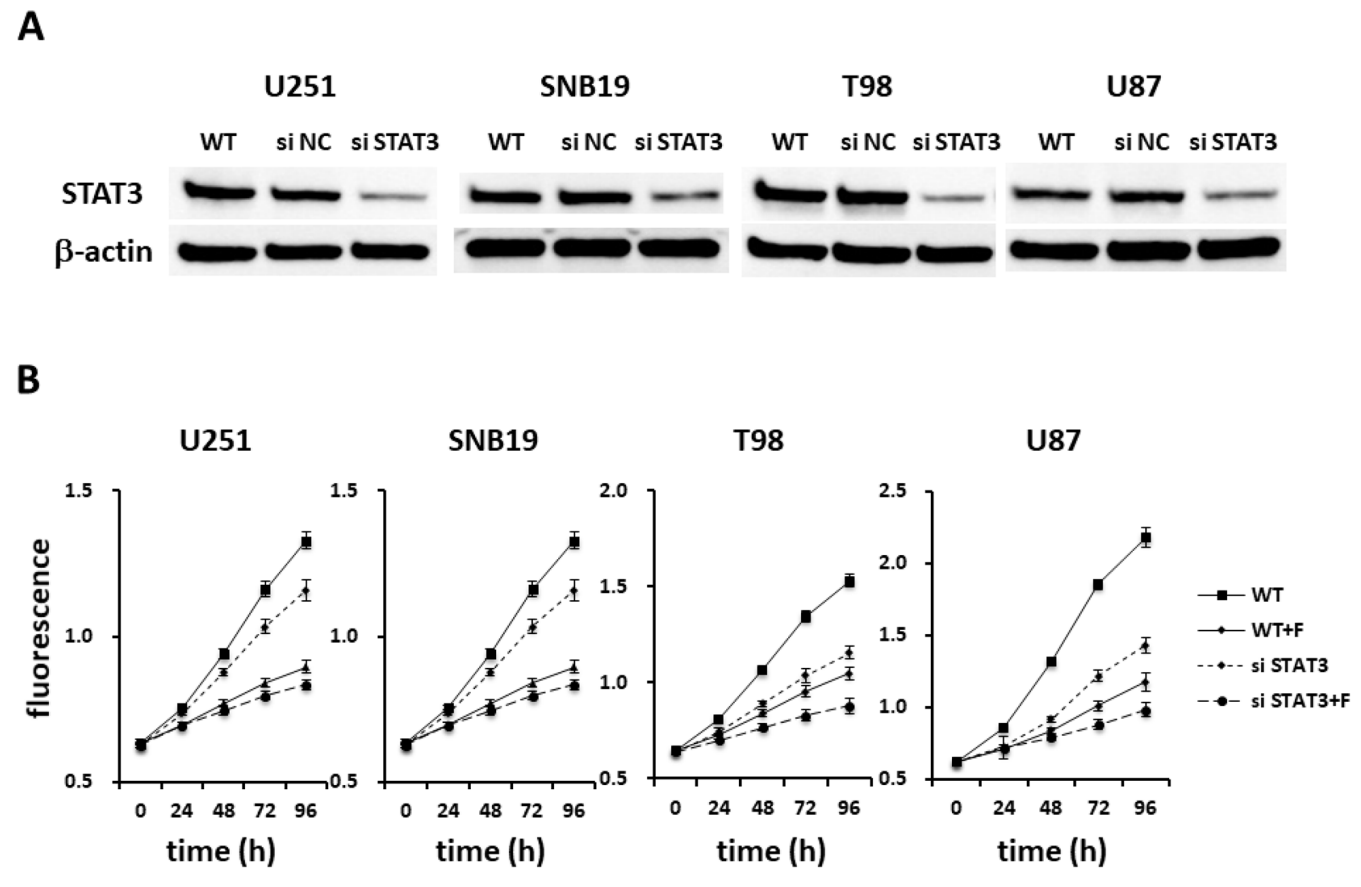
Fluspirilene as a potential STAT3 inhibitor **(A)** Extracts of U251, SNB19, T98, and U87 cells treated by siRNA for STAT3 or negative control (NC) were immunoblotted with antibodies against STAT3 or β-actin. **(B)** The viability of the GBM cells treated with fluspirilene (F) and STAT3 siRNA was significantly reduced, whereas little difference was found between STAT3 siRNA and the combination of STAT3 siRNA and fluspirilene on the cell viability.

In order to confirm whether anti-tumor effect of fluspirilene is due to only STAT3 inhibition, Alamar blue proliferation assay was performed using fluspirilene, STAT3 siRNA, and their combination. Reduction of STAT3 protein expression by siRNA was illustrated in all tested GBM cells compared with untransfected and negative control (NC) siRNA-transfected cells (Figure 5A). Fluspirilene showed little additive effect on the cell viability in combination with STAT3 siRNA treatment (Figure 5B).

**Figure 6:**
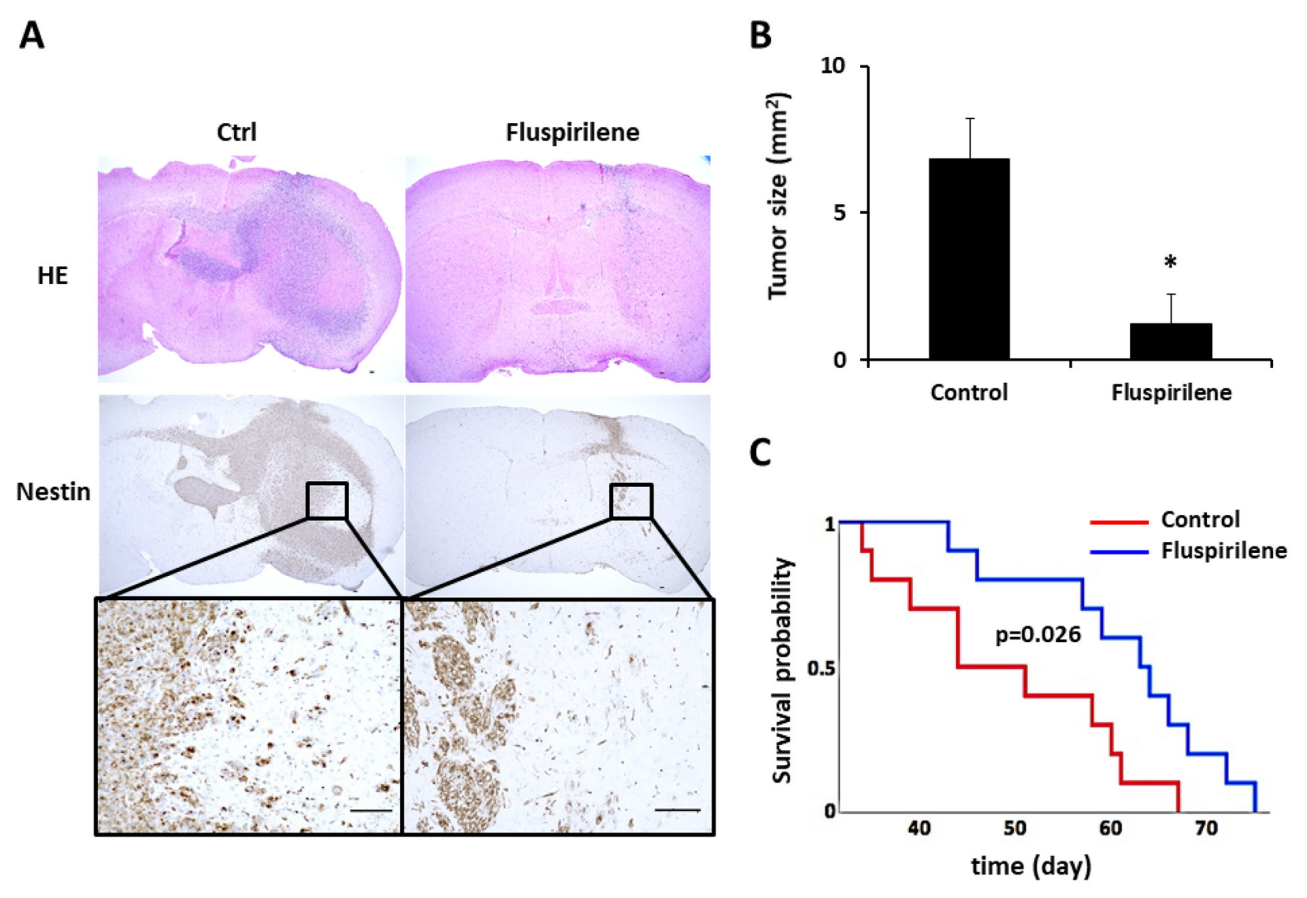
Effect of fluspirilene treatment on the GBM animal model **(A)** Representative histologic and immunohistochemical sections of untreated and fluspirilene-treated brain tumors. Tumor cells were detected by staining for nestin. A magnified image of the inset is shown below the nestin panel. Mice treated with fluspirilene showed a well-demarcated border between the tumor and adjacent normal brain tissue. *Scale bar* = 100 μm. **(B)** Mice treated with fluspirilene showed a remarkable reduction of the tumor size compared with that in the control group. ^*^p = 0.017. **(C)** Survival of mice treated with fluspirilene and DMSO (control). Fluspirilene significantly prolonged survival of the TGS04 mouse model. Log-rank test, *p* = 0.026.

**Figure 7:**
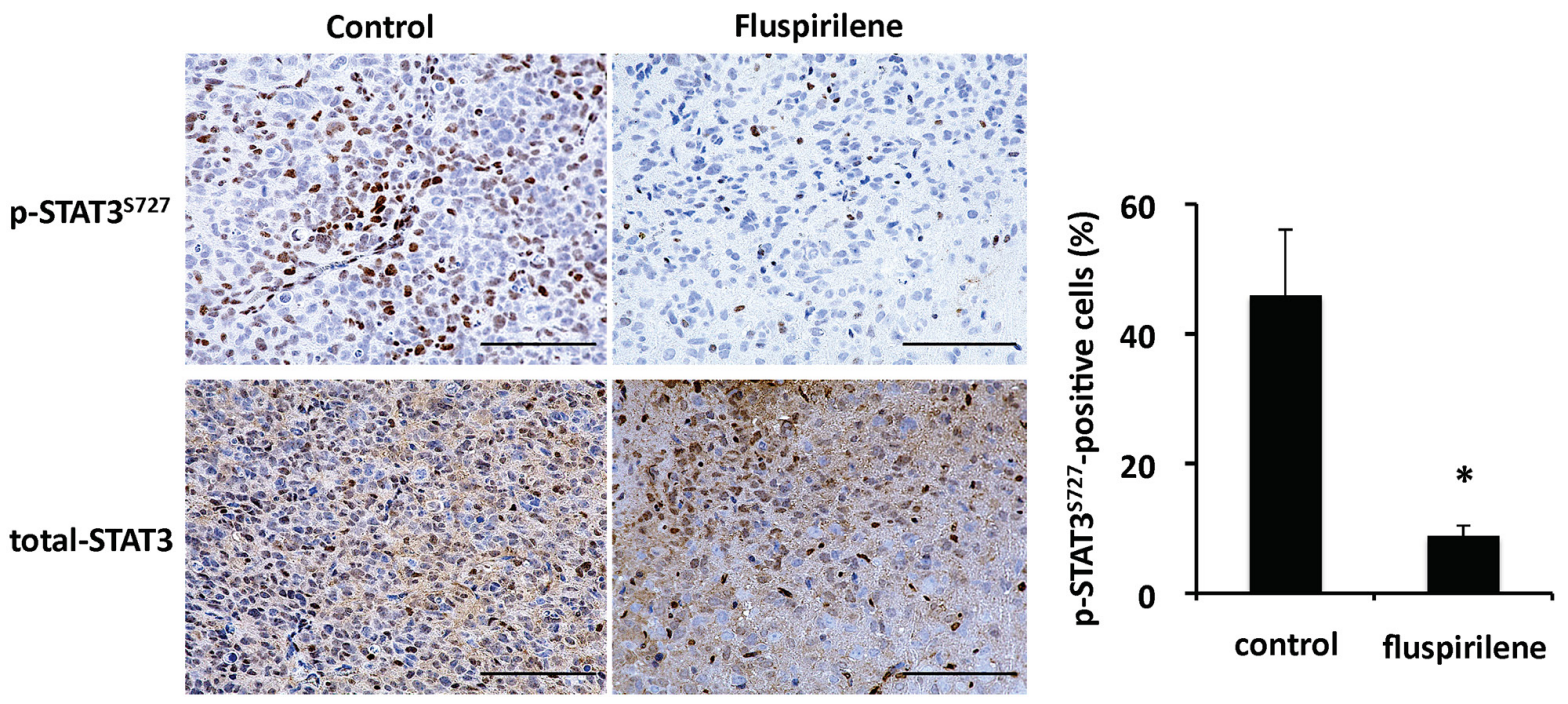
Inhibition of STAT3 activity by fluspirilene in mouse brain tumor Representative immunohistochemical sections of untreated and fluspirilene-treated brain tumors. Activity of STAT3 [level of p-STAT3 (S727) in nuclei] decreased in the mice treated with fluspirilene compared with that in the control group. ^*^p < 0.05. Scale bar = 100 μm.

Based on the results of the *in vitro* study and following an institutional review board- approved protocol, we conducted an *in vivo* tumor-forming assay. The tumor histology of our animal model demonstrated several features characteristic of host GBM, including a highly proliferative and invasive nature (Supplementary Figure 1B). The number of diffusely infiltrating satellite lesions that were stained positive for nestin significantly decreased in the fluspirilene-treated mice (Figure 6A). Notably, well-demarcated borders were observed between the tumor and adjacent normal brain tissues (Figure 6A). Moreover, the mice treated with fluspirilene showed a remarkable reduction of the tumor size compared with that in the control group (Figure 6B). Notably, fluspirilene significantly prolonged survival of the mouse model (Figure 6C). Inhibition of the STAT3 activity by fluspirilene was also confirmed by a decreased level of p-STAT3 (S^727^) in mouse brain tumor (Figure 7). These *in vivo* data were consistent with the *in vitro* results.

## DISCUSSION

In the present study, we demonstrated that fluspirilene, selected from five drug libraries composed of 1,301 compounds, suppressed the proliferation and invasion of patient-derived GSCs, glioma cells, and mouse brain tumor via inactivation of STAT3, suggesting that fluspirilene has a novel therapeutic potential against GBM as a repositioned drug. Fluspirilene, one of classic antipsychotic drugs, inhibits dopamine D2 receptor and sedates the positive symptoms of schizophrenia. This drug is administered by intramuscular injection as a suspension in water and acts sustainedly.

It has been well established that effective elimination of cancer stem cells is a key in the treatment of malignant tumors, including GBM. Previously, Gupta and colleagues [22] have established a drug screening system to select specific inhibitors of breast cancer stem cells using a chemical library including about 16,000 compounds. In this study, we identified fluspirilene as an agent inhibiting viability of GSCs and differentiated GBM cells, which demonstrated that our drug screening system was practical for the isolation of small-molecule compounds for the treatment of GBM. Tsukamoto et al. designed a screening system for a unique compound library, containing nucleosides, sugars, peptides, kinase inhibitors, and natural products, using a 3-(4,5-dimethylthiazol-2-yl)-2,5-diphenyltetrazolium bromide (MTT) assay [23]. We adopted a screening system using existing drug libraries. Hegazy et al found several drugs that suppressed the mitochondrial activity of GSCs using this system [24].

Repositioning of existing drugs has drawn attention as a promising drug discovery approach for GSC-targeted therapy. Among potential drug candidates for repurposing antipsychotics, mood stabilizers, anticonvulsants, and small molecules that can easily pass through BBB may be useful for treating GBM [25]. A number of antipsychotic drugs, mood stabilizers, and antiepileptic drugs have shown antitumor effects [26]. These drugs with the inherent ability to penetrate BBB have shown the anti-GBM potential through different mechanisms, such as the inhibition of actin polymerization by fluvoxamine [27], stimulation of AMP-activated protein kinase by olanzapine [28, 29], inhibition of histone deacetylases by valproate [30–34], and inhibition of glycogen synthase kinase 3 beta (GSK3β) by lithium [35–40]. Several small-molecule compounds have shown an anti-glioma potential. Thus, nitroxoline, an FDA-approved antibiotic, induced the apoptosis via caspase-3 [41], and disulfiram, used for the treatment of substance abuse, has shown various inhibitory effects on cancer stem cell signals [42]. Many drugs face economic barriers, such as being off-patent, which may discourage pharmaceutical companies from getting involved. Moreover, not all drugs succeed in the redevelopment, although phase I trials can be omitted. Thus, systematic screening using drug libraries, as in this study, is necessary.

STAT3 has been characterized as one of the major drivers of GBM, contributing to the maintenance of stemness and a mesenchymal phenotype via the IL-6/glycoprotein 130 (GP130)/STAT3 or EGF receptor (EGFR)/JAK2/STAT3 axis and leading to chemo- and radiation resistance of the tumor [43–45]. It has been shown that inhibition of STAT3 activation decreased the cell proliferation and invasion and enhanced the sensitivity to chemotherapy [13, 46]. In this study, fluspirilene was found to inhibit STAT3 activation, affecting not only GSCs but also GBM cells. However, it was not elucidated whether fluspirilene inhibits the STAT3 activity directly or indirectly, by acting on its upstream signals. Leigens and colleagues [47] have confirmed that metformin directly inhibited the STAT3 phosphorylation *in vitro*, using GSCs with altered STAT3 expression. Inhibition of IL-6 has been found to decrease the level of STAT3 phosphorylation, followed by downregulation of cyclin D1 [48]. Transcriptional activity of STAT3 is regulated by phosphorylation of the S^727^ residue via MAPK signaling or the PI3K/AKT/mechanistic target of rapamycin (mTOR) axis [49]. Involvement of the MEK/ERK/STAT3 pathway has been reported to suppress the ultraviolet B-induced apoptosis in melanoma [50]. In our study, declines in the levels of phosphorylated proto-oncogene tyrosine-protein kinase MDM2, CDK2, Akt, ERK, SRC, c-Jun N-terminal kinase (JNK), and p38 kinase were observed in some cell lines (Supplementary Figure 2), suggesting that the efficacy of fluspirilene varied among individual cases with different molecular profiles. SRC is activated by EGFR and induces the gene expression of factors necessary for the maintenance of GSCs, such as SOX1, C–X–C chemokine receptor type 4 (CXCR4), distal-less (DLL) protein, and neurogenic locus notch homolog protein 3 (NOTCH3), through the phosphorylation of STAT3 [51]. Fluspirilene treatment exhibited anti-tumor activity for hepatocellular carcinoma by inhibiting CDK2 [52] and induction of autophagy [53]. However, fluspirilene did not enhance the siSTAT3 treatment on cell viability (Figure 5). Taken together, it is speculated that STAT3 inhibitory potential of fluspirilene can be a major mechanism of anti-tumor effect in GBM.

A small number of drugs targeting STAT3 are under development. Three phase 1 studies reported limited antitumor effect due to common but dose-limiting adverse events such as gastrointestinal disorder, myelosuppression, peripheral neuropathy, and fatigue [18, 54, 55]. Peripheral neuropathy might not reflect cumulative toxicity because of its early onset compared with other forms of chemotherapy-induced neuropathy. Nerve growth factor- induced neurite outgrowth occurs via STAT3 activation in mitochondria not in nuclei [56], suggesting on-target effect of STAT3 inhibitors. Currently, ruxolitinib, a JAK1/2-targeting agent, is widely used for myelofibrosis and polycythemia vera [57, 58] and showed suppression of phosphorylated STAT3 in dose dependent manner [59]. Since there are many problems to be solved in the development of STAT3 inhibitory treatment, repurposing of the existing drug fluspirilene is promising as a STAT3-targeted therapy that can be introduce in clinic immediately.

JNK and p38 are downstream signaling molecules of TGFβ and mixed-lineage kinase (MLK) whose expression is enhanced in the mesenchymal subtype [14, 60, 61]. It was suggested that MAPK signaling related to TGFβ was involved in the STAT3 suppression by fluspirilene. On the other hand, fluspirilene might act specifically on STAT3 since it caused little changes in the molecular expression other than that of STAT3. Thus, we plan to focus on TGFβ and related MAPK systems as well as on the alteration of signaling necessary for the maintenance of stemness.

In conclusion, fluspirilene is a promising medicine, which not only suppresses GSCs but also shows effects on differentiated GBM cells. GBM is thought to be a mixture of GSC, its differentiated cells, reactive astrocyte, tumor-associated microglia/macrophage (TAM), etc. Thus, new drug or strategy for GBM treatment expected to have multi-acting potential against these components. There are, however, many aspects to be further clarified, such as detailed signal analysis of antitumor effects related to STAT3. In the future, to ascertain the effects of fluspirilene for drug repurposing, additional preclinical studies are required to confirm that fluspirilene contributes beneficially to the GBM treatment. Finally, clinical studies are needed to validate the efficacy and safety of fluspirilene, in combination with temozolomide and/or irradiation, for patients with GBM.

## MATERIALS AND METHODS

### Cell culture

Human GBM cell lines, U251, SNB19, T98, and U87 were purchased from American Type Culture Collection (Manassas, WA, USA) in 2009. These cell lines were characterized at the Resource Institute by short tandem repeat profile analysis. Authentication of the cell lines was unnecessary because they were expanded by culturing them for fewer than two passages and stored at −80°C. Low-passage cells were used for experiments within a period of 6 months after resuscitation. Cells were cultured at 37°C in a 5% CO_2_ incubator and maintained in Dulbecco’s modified Eagle’s medium (DMEM) supplemented with 10% heat-inactivated fetal bovine serum (FBS).

Among human patient-derived GBM cell lines, TGS01 and TGS04 were established at University of Tokyo [62], and KGS01 was established at Kanazawa University. The use of these human materials and the protocols were approved by the Ethics Committees of Kanazawa University and University of Tokyo. TGS01 and TGS04 have already been confirmed as tumor-initiating cells since cultured cells have the self-renewal ability *in vitro* and recapitulate the original tumor in a mouse xenograft model [62]. KGS01 was also confirmed as a GSC line. Briefly, KGS01 had the ability to form spheres and express surface markers characteristic of stemness, such as CD133 [1], CD44 [63], and nestin [64, 65] (Supplementary Figure 1A). KGS01 could differentiate into glial fibrillary acidic protein (GFAP)- and oligodendrocyte transcription factor (Olig2)-positive astrocyte-like cells as well as into neuron-specific class III beta-tubulin (Tuj1)-positive neuron-like cells in DMEM supplemented with 10% FBS (Supplementary Figure 1A) and grow into brain tumor, histologically recapitulating features of the original *in vivo* GBM (Supplementary Figure 1B). These cells were cultured in neurosphere medium containing DMEM/F12 (Gibco, Life Technologies, Carlsbad, CA, USA) supplemented with recombinant human EGF at 20 ng/mL (Sigma–Aldrich, St. Louis, MO, USA), recombinant human basic fibroblast growth factor at 20 ng/mL (Sigma–Aldrich), B27 supplement without vitamin A (Gibco), and GlutaMAX (Gibco).

### Drug screening

We used the following five SCREEN-WELL® libraries from Enzo Life Sciences (Farmingdale, NY, USA): 1) FDA (U.S. Food and Drug Administration)-approved drug library (640 compounds; CB-BML-2841J0100); 2) ICCB (Harvard Institute of Chemistry and Cell Biology) known bioactives library (480 compounds; CB-BML-2840J0100); 3) kinase inhibitor library (80 compounds; CB-BML-2832J0100); 4) fatty acid library (68 compounds; CB-BML-2803J0100); and 5) phosphatase inhibitor library (33 compounds; CB-BML-2834J0100). To identify effective compounds among the 1,301 compounds, three-step screening was performed (Figure 1). First, the three GSC lines (TGS01, TGS04, and KGS01) were treated with each compound provided in the libraries at three concentrations (1, 5, and 20 μM) in a 384-well plate (Corning, Cambridge, MA, USA) for 48 h, followed by a 2-(2-methoxy-4-nitrophenyl)-3-(4-nitrophenyl)-5-(2,4-disulfophenyl)-2*H*-tetrazolium (WST-8) cell viability assay as described below. Compounds exhibiting 25% or more reduction in cell viability in a dose-dependent manner were selected. Second, drugs that have already been reported to have a therapeutic potential against GBM or those in clinical trials were excluded. Third, the WST-8 assay and sphere-forming assay were performed using the three GSC lines with lower concentrations (0.2, 0.5, and 1 μM) of the selected compounds. Candidate drugs were identified as demonstrating remarkable inhibition of cell viability even at the lowest concentration.

### Cell viability assay

GSC viability was assessed using a Cell Counting Kit-8 (Dojindo, Kumamoto, Japan) following the manufacturer’s instructions. Briefly, GSC spheres were dissociated into single cells with StemPro Accutase (Gibco). Then, the cells were seeded into a 96-well Costar ultra-low attachment plate (Corning) at a density of 1 × 10^3^ cells/100 μL. The relative numbers of viable cells were determined by measuring the absorbance with the WST-8 (2-(2-methoxy-4-nitrophenyl)-3-(4-nitrophenyl)-5-(2,4-disulfophenyl)-2H-tetrazolium) assay kit using a microplate reader.

An Alamar blue assay (Biosource, Camarillo, CA, USA) was performed to examine the viability of the GBM cell lines. According to the manufacturer’s manual, 1 × 10^3^ GBM cells of each type were seeded into wells of a 96-well plate in 200 μL of culture medium supplemented with 0.1% FBS. After 4 h of incubation at 37°C, 20 μL of Alamar blue was added to each well. The plate was read on a fluorescence plate reader at 0, 24, 48, 72, and 96 h. The average fluorescence values from eight wells were calculated and plotted. To investigate the effect of fluspirilene on cell proliferation, cells were treated with various concentrations of fluspirilene.

### Tumor sphere-forming assay

The sphere-forming assay was performed as described previously [66, 67]. Briefly, GSC spheres were dissociated into single cells with StemPro Accutase (Gibco). Then, 1 × 10^3^ single cells were seeded in a 96-well Costar ultra-low attachment plate (Corning) in 200 μL of neurosphere medium supplemented with 1.0% methylcellulose. The cells were treated with either fluspirilene (Sigma–Aldrich) or dimethyl sulfoxide (DMSO). The numbers of tumor spheres were counted, and the tumor sphere diameters were measured after 7 days of incubation.

### Matrigel invasion assay

Cell invasion assays were performed using modified Boyden chambers consisting of Transwells with Matrigel-pre-coated membrane filter inserts in 24-well tissue culture plates (BD Biosciences, San Jose, CA, USA), as described previously [68]. Serum-deprived GBM cells were suspended in DMEM supplemented with 0.1% FBS and added to each Transwell. GSC spheres were dissociated into single cells with StemPro Accutase (Gibco). GSCs were suspended in F12/DMEM supplemented without growth factor and added to each Transwell. After 16–20 h of incubation at 37°C, the non-invasive cells were removed by wiping the upper side of the filter, and the invasive cells were fixed with methanol and stained using a Diff-Quik kit (Sysmex, Kobe, Japan). The invaded cells were counted on the filter in six microscopic fields randomly selected at a high power, and the mean number of invasive cells was calculated and analyzed.

### Western blot analysis

Cellular proteins were extracted from cultured cells following the treatment with lysis buffer (Sigma–Aldrich) containing a mixture of protease and phosphatase inhibitors (Sigma–Aldrich). A 15-μg aliquot of the whole protein extract was analyzed by a western immunoblot assay for the protein of interest (Supplementary Table 1), as previously described [68].

### Silencing of endogenous STAT3 with small interfering RNA

Purified duplexed small interfering RNA (siRNA) for STAT3 and control luciferase were purchased from QIAGEN. Ten nanomolar siRNA was transfected into cells cultured in 6-well plate using Avalanche®-Omni Transfection Reagent (APRO SCIENCE, Tokushima, Japan). Transfected cells were cultured for 48 h before use.

### Mouse model of GBM and treatment

Following an institutional review board-approved protocol, we generated a mouse brain tumor model of human GBM by transplantation of TGS04 cells into the brain of nude mice (BALB/cSlc-nu/nu, Charles River Laboratories, Osaka, Japan) according to our previous study [69]. Briefly, a burr hole was made in the skull 3 mm lateral to the bregma using a drill, and 3 × 10^4^ TGS04 cells were stereotactically injected at a depth of 3 mm below the dura mater. The mice were randomly assigned to two groups and treated with either fluspirilene (n = 5) or with DMSO as a control group (n = 5). All mice were given intramuscular injections of 200 μL of DMSO or fluspirilene dissolved in DMSO at 1 mg/kg body weight four times. The dose of fluspirilene corresponded to the concentration of the drug in culture medium used in the treatment of cells *in vitro*. Assuming that 60% of body weight is accounted for by body fluid in each mouse, the dose of fluspirilene of 1mg/kg corresponded approximately to concentration of 5 μM in culture medium. After 6 weeks of treatment, all ten mice were euthanized.

The brain tissues were dissected, embedded in paraffin, and then cut into 4-μm serial coronal sections. The tissue sections were stained using a standard hematoxylin and eosin staining technique. We calculated the surface included by the tumor contour of the region of interest in coronal section showing the maximal area of each tumor. For estimates of overall survival time, the mice were randomly assigned to two groups for treatment with fluspirilene (n = 10) and with DMSO as a control group (n = 10). All animal experiments followed the Guidelines for the Care and Use of Laboratory Animals at Kanazawa University that covers the national guideline.

### Immunohistochemistry and immunofluorescence assay

For immunohistochemistry, 4-μm thick paraffin-embedded tissue blocks were sectioned onto slides and deparaffinized. The sections were microwaved for 15 min in citrate buffer (pH 6.0), quenched with 0.3% hydrogen peroxide (H_2_O_2_) in methanol for 20 min, and blocked for 20 min with skim milk. The slides were incubated with each primary antibody (Supplementary Table 1) or with nonimmune mouse or rabbit IgG as a negative control overnight at 4°C, then washed, and immunostained using an Envision+ kit (Dako, Tokyo, Japan) for 60 min at room temperature. The color was developed using 3,3′^-^diaminobenzidine tetrahydrochloride for 5 min, and the sections were then counterstained with hematoxylin.

For immunofluorescence assay, cells were fixed with 4% paraformaldehyde and then permeabilized. After washing with phosphate-buffered saline, the cells were blocked with 5% skim milk for 30 min and incubated with each antibody (Supplementary Table 1) for 1 h at 25°C, followed by incubation with an Alexa Fluor 488 goat anti-rabbit antibody or an Alexa Fluor 546 anti-mouse antibody for 1 h at room temperature in the dark. Finally, the sections were washed with Tris-buffered saline containing Tween 20 and mounted with mounting medium for the fluorescence assay with 4′,6-diamidino-2-phenylindole (Santa Cruz Biotechnology, CA, USA). Images were acquired using a BZ-X700 microscope (Keyence, Osaka, Japan) and digitally processed with the Keyence analysis software (Keyence).

### Statistical analysis

The data are presented as the mean ± standard deviation. Statistically significant differences in the mean or median values between the two groups were tested by the Student’s *t*-test or Mann–Whitney *U*-test as appropriate. Log-rank analysis was used to determine statistical significance of Kaplan-Meier survival curve. A value of *p* < 0.05 was considered to indicate a statistically significant difference. All statistical calculations were performed using the SPSS software, version 19.0 (SPSS, Inc., Chicago, IL, USA).

## SUPPLEMENTARY MATERIALS FIGURES AND TABLE


