# Supplementary material for: Identification of antipsychotic drug fluspirilene as a potential anti-glioma stem cell drug
Source: Oncotarget. 2017 Dec 4;8(67):111728–41. doi: 10.18632/oncotarget.22904 (PMC5762355; doi:10.18632/oncotarget.22904)
Supplement: Supplementary file 1 [file oncotarget-08-111728-s001.pdf]

Identification of antipsychotic drug fluspirilene as a potential anti-glioma stem cell drug

SUPPLEMENTARY MATERIALS

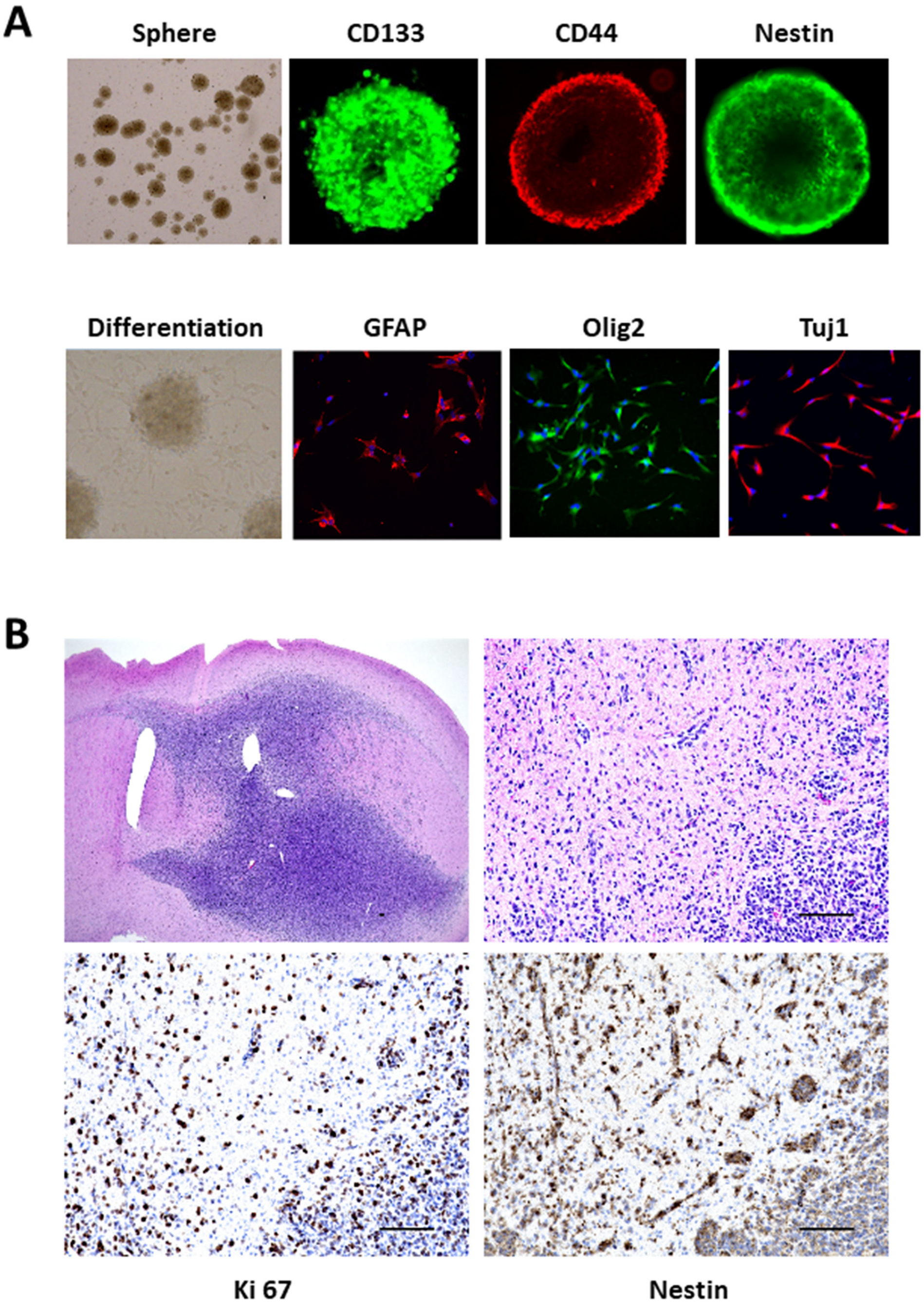

Supplementary Figure 1: Stemness, differentiation, and tumor-initiating potential of KGS01. **(A)** Tumorspheres derived from a GBM surgical specimen (KGS01) were positively stained for CD133, CD44, and nestin, which are the characteristic markers of mesenchymal-type stem cells. Magnification, ×400. Tumorspheres differentiated into GFAP- and Olig2-positive astrocyte-like cells and Tuj1-positive neuron-like cells in DMEM supplemented with 10% FBS. Magnification, ×200. **(B)** KGS01 cells (5×10^4) were transplanted into the mouse brain, and a brain tumor histologically recapitulating the features of original GBM developed 60 days later. Tumor cells were positive for nestin and showed a high antigen Ki -67 staining index. *Scale bar* =100 µm.

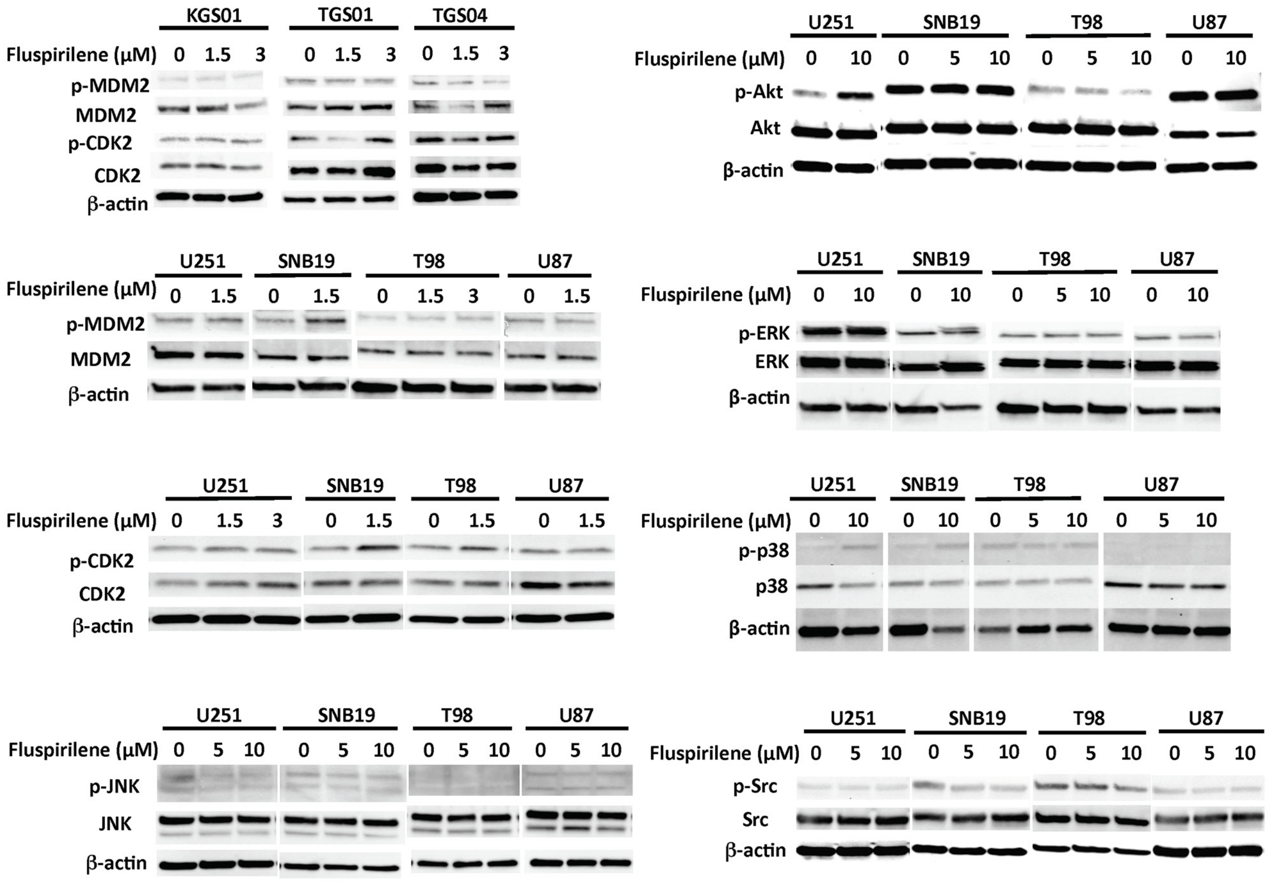

Supplementary Figure 2: Western blot analysis of pathways targeted by fluspirilene. Fluspirilene down-regulated phosphorylation or expression level of MDM2, CDK2, Akt, ERK1/2, p38, JNK and Src in some GSCs or GBM cells.

Supplementary Table 1: Source and working dilutions of the primary antibodies used for Western blotting, immunohistochemical staining, and immunofluorescence

Antibody	MW (kDa)	Source	Working dilution	Company
Akt	60	Rabbit	1:1000 (WB)	Cell Signaling
p-Akt (S^{473})	60	Rabbit	1:1000 (WB)	Cell Signaling
β-actin	42	Mouse	1:5,000 (WB)	WAKO
CD44	29-37	Mouse	1:400 (IF)	DAKO
CD133	97	Mouse	1:400 (IF)	R&D Systems
CDK2	33	Rabbit	1:1000 (WB)	Cell Signaling
p-CDK2 (T^{160})	33	Rabbit	1:1000 (WB)	Cell Signaling
ERK1/2	44, 42	Rabbit	1:1000 (WB)	Cell Signaling
p-ERK1/2 (T^{202}/Y^{204})	44, 42	Rabbit	1:1000 (WB)	Cell Signaling
GFAP	55	Rabbit	1:600 (IF)	DAKO
JNK	46, 54	Rabbit	1:1000 (WB)	Cell Signaling
p-JNK (T^{183}/Y^{185})	46, 54	Rabbit	1:500 (WB)	Cell Signaling
Ki-67	359	Rabbit	1:800 (IHC)	Thermo Scientific
MDM2	90	Rabbit	1:1000 (WB)	NOVUS
p-MDM2 (S^{166})	90	Rabbit	1:500 (WB)	Cell Signaling
Nestin	260	Mouse	1:400 (IHC), 1:200 (IF)	BD Biosciences
Olig2	32	Rabbit	1:500 (IF)	IBL
p38	40	Rabbit	1:1000 (WB)	Cell Signaling
p-p38 (T^{180}/Y^{182})	43	Rabbit	1:1000 (WB)	Cell Signaling
SOX2	34	Rabbit	1:2000 (WB)	Gene Tex
Src	60	Rabbit	1:1000 (WB)	Cell Signaling
p-Src (Y^{416})	60	Rabbit	1:1000 (WB)	Cell Signaling
STAT3	80	Mouse	1:2,000 (WB), 1:500 (IHC), 1:400(IF)	Cell Signaling
p-STAT3 (S^{727})	80	Rabbit	1:500 (WB), 1:200 (IHC)	Cell Signaling
Tuj1	55	Mouse	1:200 (IF)	R&D system

Abbreviations: CDK2, cyclin-dependent kinase 2; ERK1/2, extracellular signal-regulated kinase 1/2; GFAP, glial fibrillary acidic protein; IF, immunofluorescence; IHC, immunohistochemistry; JNK, c-Jun N-terminal kinase; MDM2, murine double minute 2; MW, molecular weight; SOX2, SRY (sex determining region Y)-box 2; STAT3, signal transducer and activator of transcription 3; WB, Western blotting
